# Syndromic Management and STI Control in Urban Peru

**DOI:** 10.1371/journal.pone.0007201

**Published:** 2009-09-25

**Authors:** Jesse L. Clark, Andres G. Lescano, Kelika A. Konda, Segundo R. Leon, Franca R. Jones, Jeffrey D. Klausner, Thomas J. Coates, Carlos F. Caceres, for the NIMH International Collaborative HIV/STD Prevention Trial

**Affiliations:** 1 Division of Infectious Diseases and Program in Global Health, David Geffen School of Medicine at the University of California, Los Angeles, California, United States of America; 2 U.S. Naval Medical Center Research Detachment, Lima, Peru; 3 Unidad de Salud Sexual y Derechos Humanos, Facultad de Salud Publica, Universidad Peruana Cayetano Heredia, Lima, Peru; 4 Naval Medical Research Center, Biological Defense Research Directorate, Silver Spring, Maryland, United States of America; 5 San Francisco Department of Public Health, San Francisco, California, United States of America; UCL Institute of Child Health, United Kingdom

## Abstract

**Background:**

Syndromic management is an inexpensive and effective method for the treatment of symptomatic sexually transmitted infections (STIs), but its effectiveness as a method of STI control in at-risk populations is questionable. We sought to determine the potential utility of syndromic management as a public health strategy to control STI transmission in high-risk populations in urban Peru.

**Methodology:**

We surveyed 3,285 at-risk men and women from three Peruvian cities from 2003–05. Participants were asked about the presence of genital ulcers, discharge, or dysuria in the preceding six months. Participants reporting symptoms were asked about subsequent health-seeking and partner notification behavior. Urine and vaginal swab samples were tested for *Neisseria gonorrhoeae* and *Chlamydia trachomatis* by nucleic acid testing. Serum was tested for syphilis and Herpes Simplex Virus-Type 2 antibodies.

**Findings:**

Recent urogenital discharge or dysuria was reported by 42.1% of participants with gonorrhea or chlamydia versus 28.3% of participants without infection. Genital ulceration was reported by 6.2% of participants with, and 7.4% of participants without, recent syphilis. Many participants reporting symptoms continued sexual activity while symptomatic, and approximately half of all symptomatic participants sought treatment. The positive and negative predictive values of urogenital discharge or genital ulcer disease in detecting STIs that are common in the study population were 14.4% and 81.5% for chlamydia in women and 8.3% and 89.5% for syphilis among gay-identified men.

**Conclusions:**

In our study, STIs among high-risk men and women in urban Peru were frequently asymptomatic and symptomatic participants often remained sexually active without seeking treatment. Additional research is needed to assess the costs and benefits of targeted, laboratory-based STI screening as part of a comprehensive STI control program in developing countries.

## Introduction

Syndromic management is a simple and effective method for the treatment of symptomatic sexually transmitted infections (STIs) in resource-limited settings [1]–[6]. Several studies have demonstrated improvements in clinical STI management following training for healthcare professionals using World Health Organization guidelines for syndromic treatment of urethritis and genital ulceration [6]–[8]. However, in many developing countries, limited healthcare resources also lead to a reliance on syndromic management as the primary method for the detection and treatment of STIs in the population. Despite its utility in the treatment of symptomatic infection, little data is available concerning the effectiveness of syndromic management as a strategy to control STI transmission in resource-limited environments [9]–[12].

In Peru, as in many developing countries, standard clinical approaches to urethritis, genital ulcer disease (GUD), cervicitis, and pelvic inflammatory disease are based on syndromic management principles. According to practice guidelines issued by the Peruvian Ministry of Health, patients presenting with signs or symptoms of dysuria, urethral discharge, vaginal discharge, or cervicitis should be treated presumptively for both gonorrhea and chlamydia [13]. Patients who are found to have GUD on physical examination or who report a recent history of a spontaneously resolving genital ulcer should be screened with a Rapid Plasma Reagin (RPR) assay and, if reactive, treated for both syphilis and chancroid. (For ulcerative lesions with a vesicular appearance, the guidelines also recommend empiric acyclovir treatment.) While men who have sex with men (MSM), sex workers and pregnant women are recommended to undergo routine serologic testing for HIV and syphilis, screening procedures for other STIs, including gonorrhea and chlamydia, and in other at-risk populations are based primarily on a syndromic approach.

We sought to assess the utility of syndromic management as a public health strategy to control STI transmission in high-risk populations in urban Peru. We analyzed previously collected data from a sample of at-risk men and women in three coastal Peruvian cities to quantify the prevalence of symptomatic and asymptomatic STIs, the participant's subsequent response to symptoms, and the public health implications of a primarily syndromic approach to STI control.

## Methods

### Study Design

As part of an international HIV/STI prevention trial, we conducted an epidemiological survey of low-income populations from three coastal cities in Peru between 2003 and 2005. Baseline surveys were completed in Lima in 2003 and in the cities of Trujillo and Chiclayo in 2005. A total of 20 low-income urban communities (*barrios*) in the three cities were identified for participant recruitment. Three subpopulations at risk for HIV and STI transmission were identified during ethnographic analysis of the *barrios*: gay-identified men who have sex only with men, heterosexual-identified men who engage in sex with multiple partners that may include other men, and sexually active heterosexual-identified women. Detailed information concerning the identification and recruitment of the three high-risk subpopulations has been published previously [14], [15]. Briefly, men and women from these subpopulations were eligible for enrollment if they were 18–40 years old, planned to remain in the area for the entire length of the study (two years), and regularly visited areas of high-frequency social interaction in the *barrio* (*e.g.,* soccer fields, hair salons, and street corners). Approximately 150 eligible individuals were recruited from each *barrio*. All respondents who agreed to participate provided written informed consent. The study protocol was approved by the Committee of Human Subjects Research of the University of California at Los Angeles, the Universidad Peruana Cayetano Heredia, and the U.S. Naval Medical Research Center in Bethesda, MD in compliance with all federal regulations regarding the protection of human subjects.

### Data Collection

Participants were interviewed in a temporary project office located in their *barrio* with a Computer Assisted Personal Interviewing system. Trained staff read participants questions in Spanish and entered the responses directly into a secure computer database. Participants were assured of the confidentiality of all of their responses. All participants were asked whether they had experienced dysuria or “pain when you urinated” (“*te dolió al orinar*”), penile or vaginal “discharge or secretions,” (“*descarga uretral o secreciones*”), or a “genital ulcer” (“*úlcera genital*”) on their penis or vagina within the previous six months (there were no questions concerning anorectal symptoms). An affirmative response to any of these questions prompted a series of follow-up questions about actions taken following the onset of symptoms including whether they did anything to prevent infecting their sex partner(s), whether they did anything to cure themselves of the symptoms and, if so, what specific actions were taken. All participants were asked the same questions, regardless of gender or reported sexual behavior.

Study interviewers provided pre-test counseling and phlebotomy staff collected blood samples from all participants. Women were asked to self-collect a vaginal swab specimen. All men, and any women who declined to collect a vaginal swab, were asked to provide a urine sample. Approximately one month after the initial evaluation, participants returned for post-test counseling and provision of results. Participants with a curable STI were provided with appropriate antibiotic therapy. Participants with symptomatic Herpes Simplex Virus Type 2 infection were treated with acyclovir. Participants diagnosed with HIV infection were referred to specialized Ministry of Health treatment centers for ongoing care. All participants diagnosed with an STI were advised of the importance of partner notification and were offered free partner testing and treatment at the study site.

### Laboratory Methods

Biological samples were processed at the U.S. Naval Medical Research Center Detachment in Lima, Peru. Urine and vaginal swab specimens were analyzed for the presence of *Neisseria gonorrhoeae* (GC) and *Chlamydia trachomatis* (CT) with nucleic acid amplification testing (Amplicor, Roche Diagnostics; Alameda, CA). Blood was screened for syphilis infection by RPR assay (RPRnosticon, Biomérieux; Marcy l'Étoile, France). RPR-positive specimens were confirmed by Treponema Pallidum Particle Agglutination (TPPA) assay (Serodia, Fujirebio; Tokyo, Japan) and the RPR titer quantified. Only TPPA-positive specimens with RPR titers ≥1∶8 (suggestive of recent infection) were included in our analysis. Serum antibodies to Herpes Simplex Virus- Type 2 (HSV-2) were detected by ELISA assay (HerpeSelect, Focus Technologies; Cypress, CA), using a cut-off index value of >3.49 to define seropositive specimens.

### Data Analysis

We analyzed results using chi-square tests and Fisher's exact tests when appropriate. All p-values are two-sided and considered statistically significant if p<0.05. Stata 9.0 software was used for all analyses (Stata Corporation, College Station, TX).

## Results

### Sociodemographic Factors

A total of 3,301 participants were enrolled, of whom 3,285 provided biological samples (Table 1). Among the three sub-populations, 2,424 participants (73.8%) were heterosexual-identified men, 541 (16.4%) were homosexual-identified men, and 320 (9.7%) were heterosexual women. The age range of participants was 18–41, with a median age of 23 years (interquartile range: 20–27).

**Table 1:** Socio-demographic Factors, Reported Genitourinary Symptoms, and Prevalence of Laboratory-confirmed STIs; Lima, Trujillo, and Chiclayo, Peru; 2003–05.

		Heterosexual Men n = 2424		Homosexual Men n = 541		Heterosexual Women n = 320		Total n = 3285	
**Demographics**									
Age (years)									
	Median (IQR)^a^	22 (20–26)		26 (23–32)		25 (21–31)		23 (20–28)	
Relationship status									
	Single	1689	69.7%	511	94.5%	116	36.3%	2316	70.5%
	Primary partner	612	25.3%	27	5.0%	162	50.6%	801	24.4%
	Formerly married	122	5.0%	3	0.6%	42	13.1%	167	5.1%
Graduated high school		1163	48.0%	358	66.2%	130	40.6%	1651	50.3%
Had a child/children		738	30.5%	18	3.3%	239	74.7%	995	30.3%
Regularly earns money		2073	85.5%	443	81.9%	178	55.6%	2694	82.0%
**Prevalence of STI Symptoms**									
Dysuria		655	27.1%	96	17.8%	135	42.2%	886	27.0%
Urethral discharge		77	3.2%	9	1.7%	104	32.6%	190	5.8%
Genital ulcer		196	8.1%	24	4.4%	20	6.3%	240	7.3%
**Prevalence of Laboratory-confirmed STIs**									
Herpes Simplex Virus-2		315	13.0%	373	69.0%	130	40.6%	818	24.9%
Syphilis (Any RPR)		56	2.3%	120	22.2%	12	3.8%	188	5.7%
Syphilis (RPR ≥1∶8)		33	1.4%	57	10.5%	7	2.2%	97	3.0%
Gonorrhea^b^		11	0.5%	2	0.4%	9	2.8%	22	0.7%
Chlamydia^b^		127	5.2%	6	1.1%	45	14.1%	178	5.4%

a Interquartile range

b Genital only

### Prevalence of Genitourinary Symptoms

Among all participants, regardless of STI status, 27.0% reported symptoms of genital discharge and/or dysuria in the previous six months. Genital discharge or dysuria was reported by 42.1% of all participants with gonorrhea or chlamydia, and by 48.1% of women with gonorrhea or chlamydia. An ulcerative genital lesion during the previous six months was reported by 7.2% of all participants, by 6.2% of all participants with an RPR titer ≥1∶8, and by 3.5% of gay-identified men with an RPR titer ≥1∶8.

### Participant Response to Symptoms

**Figure 1:**
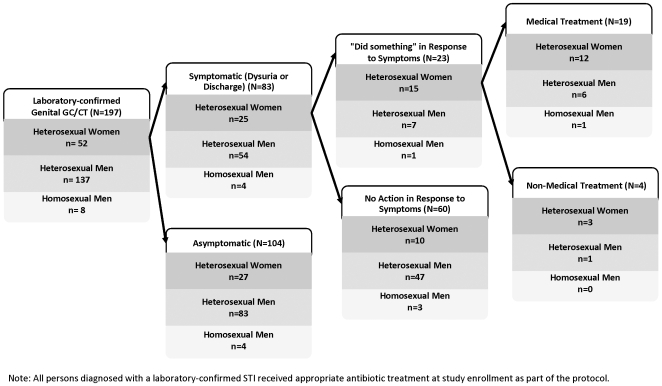
Pre-enrollment Treatment Outcomes Among Men and Women with Genital Gonorrhea and/or Chlamydia; Lima, Trujillo and Chiclayo, Peru 2003–05.

**Figure 2:**
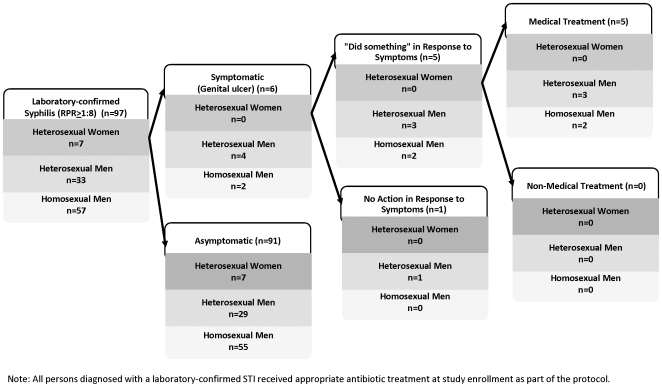
Pre-enrollment Treatment Outcomes Among Men and Women with Recent Syphilis (RPR≥1∶8); Lima, Trujillo and Chiclayo, Peru 2003–05.

The actions taken by participants in response to reported symptoms are described in Tables 2 and 3. Few differences in participant behavior were observed when analyzed according to gender, sexual identity, or type of symptoms. Although the majority of women reported notifying their sex partner(s) when they experienced genital discharge or ulceration, the majority of men, regardless of their sexual identity, did not inform their partners of any genitourinary symptoms. Among participants who reported genital discharge, 61.8% (118/191) took some action to cure their symptoms, and 49.7% (95/191) used medicine to treat their symptoms. For participants reporting genital ulceration, 66.7% (161/241) took some curative action and 48.6% (117/241) used medicine. Ultimately, 9.6% of participants with laboratory-confirmed gonorrhea or chlamydia and 5.2% of participants with recent syphilis received some form of medical treatment prior to study enrollment (Figures 1 and 2). Symptomatic participants who had sought treatment were more likely than those who did not seek treatment prior to enrollment to be diagnosed with gonorrhea or chlamydia at the time of evaluation (9.6% vs. 2.5%; p<0.001), though they were not significantly more likely to be diagnosed with syphilis (5.2% vs. 3.4%; p = 0.348). A substantial minority of symptomatic participants continued to engage in sexual activity despite the presence of genital discharge [47.6% (91/191)] or ulceration [41.1% (99/241)]. Participants who sought treatment for their symptoms were more likely to abstain from sex than those who took no protective action while experiencing genital discharge (58% vs. 44%; p = 0.063) or ulceration (64% vs. 49%; p = 0.022).

**Table 2:** Response to STI Symptoms (All Participants); Lima, Trujillo and Chiclayo, Peru; 2003–05.

		Heterosexual-identified Men	Homosexual-identified Men	Women	All
**Discharge cases (n)**		**77**	**9**	**105**	**191**
	Told partner (%)	22.1	22.2	54.3	39.8
	Abstinence (%)	39.0	77.8	51.4	47.6
	“Did something” about symptoms (%)	58.4	77.8	62.9	61.8
	Used condom (%)	5.2	11.1	4.8	5.2
	Used folk remedy (%)	9.1	11.1	26.7	8.8
	Used medicine (%)	53.3	66.7	45.7	49.7
	Other (%)	1.3	11.1	2.9	2.6
**Ulcer cases (n)**		**197**	**24**	**20**	**241**
	Told partner (%)	43.1	33.3	65.0	44.0
	Abstinence (%)	42.6	29.2	40.0	41.1
	“Did something” about symptoms (%)	68.0	62.5	60.0	66.8
	Used condom (%)	7.1	8.3	0.0	6.6
	Used folk remedy (%)	22.3	20.8	35.0	23.2
	Used medicine (%)	48.7	45.8	50.0	48.6
	Other (%)	5.6	4.2	0.0	5.0

**Table 3:** Response to STI Symptoms (Participants with Ongoing Sexual Activity); Lima, Trujillo and Chiclayo, Peru 2003–05.

	Heterosexual-identified Men	Homosexual-identified Men	Women	All
**Discharge cases (n)**	**30**	**7**	**54**	**91**
“Did something” about symptoms (%)	40.0	85.7	61.1	56.0
Used condom (%)	13.3	14.3	9.3	11.0
Used folk remedy (%)	10.0	14.3	31.5	23.1
Used medicine (%)	30.0	71.4	40.7	39.6
Other (%)	3.3	14.3	3.7	4.4
**Ulcer cases (n)**	**84**	**7**	**8**	**99**
“Did something” about symptoms (%)	64.3	57.1	50.0	62.6
Used condom (%)	16.7	28.6	0.0	16.2
Used folk remedy (%)	21.4	0.0	25.0	20.2
Used medicine (%)	44.0	28.6	50.0	43.4
Other (%)	7.1	14.3	0.0	20.2

### STI Prevalences

The prevalence of gonorrhea or chlamydia, syphilis and HSV-2 infection in the study population is reported in Table 1. Active syphilis (RPR≥1∶8) was diagnosed in 10.5% (57/541) of homosexual-identified men, 1.4% (33/2424) of heterosexual-identified men, and 2.2% (7/320) of women. Urogenital gonorrhea or chlamydia was identified in 1.5% (8/544) of homosexual men, 5.6% (137/2434) of heterosexual men and 16.2% (52/320) of women.

### Diagnostic Performance of Symptomatic Screening Criteria

The diagnostic performance of symptom recognition for STI screening in our study population is reported in Table 4. Participant-reported genital discharge and/or dysuria in the preceding six months had a positive predictive value (PPV) of 14.4% and a negative predictive value (NPV) of 81.5% for gonorrhea or chlamydia in women. Among homosexual-identified men, the PPV and NPV of self-reported recent genital ulceration for active syphilis was 8.3% and 89.5%, respectively, and 79.2% and 29.5% in screening for HSV-2 infection.

**Table 4:** Diagnostic Performance of STI Symptoms; Lima, Trujillo, Chiclayo, Peru 2003–05.

STI	Symptom	Population	STI Prevalence	Diagnostic Sensitivity	Diagnostic Specificity	PPV	NPV
**Gonorrhea and/or Chlamydia**	**Dysuria and/or Discharge**	Homosexual-identified Men	1.5%	50.0%	82.1%	4.0%	99.1%
		Heterosexual-identified Men	5.6%	39.4%	72.4%	7.9%	95.2%
		Women	16.2%	48.1%	44.4%	14.4%	81.5%
		All	6.0%	42.1%	71.7%	8.6%	95.1%
**Syphilis (RPR ≥1∶8)**	**Genital Ulcer**	Homosexual-identified Men	10.5%	3.5%	95.5%	8.3%	89.5%
		Heterosexual-identified Men	1.4%	12.1%	92.0%	2.0%	98.7%
		Women	2.2%	0.0%	93.6%	0.0%	97.7%
		All	3.0%	6.2%	92.7%	2.5%	97.0%
**Herpes Simplex Virus Type 2**	**Genital Ulcer**	Homosexual-identified Men	69.0%	5.1%	96.7%	79.2%	29.5%
		Heterosexual-identified Men	13.0%	14.3%	93.2%	24.7%	87.4%
		Women	40.6%	7.7%	94.5%	50.0%	58.9%
		All	24.9%	9.0%	93.5%	32.7%	74.7%

## Discussion

Based on our analysis of reported genitourinary symptoms, subsequent treatment-seeking behavior, and laboratory-based STI screening among high-risk men and women in urban Peru, an STI control strategy based solely on syndromic management would not be sufficient to address commonly occurring STIs in this population. In our analysis, screening criteria such as participant-reported episodes of dysuria, genital discharge, and genital ulceration had poor positive and negative predictive values when compared with laboratory-based testing for gonorrhea, chlamydia and recent syphilis in sub-populations of men and women with a high prevalence of infection. The inability of the assessed symptomatic complexes to account for asymptomatic STIs that may result in clinical morbidity and continued STI transmission was compounded by the failure of many subjects who experienced symptoms to seek medical treatment [16]–[19].

Multiple studies have demonstrated the value of syndromic management in the empiric diagnosis and treatment of patients presenting for care with symptomatic STIs. In a series of studies in Peruvian pharmacies and other healthcare settings, Garcia *et al.* found syndromic management to be an effective and inexpensive method for the clinical management of symptomatic STIs [3], [8], [20]–[22]. While our data suggests concerns related to unnecessary antibiotic use resulting from the low diagnostic specificity of syndromic management, it remains an inexpensive and readily available method for the diagnosis and treatment of symptomatic STIs in both developing and developed countries.

For subpopulations with a high burden of asymptomatic infections, including syphilis in men who have sex with men (MSM) and chlamydia in heterosexual women, our data suggest the need for routine screening of these specific, high-prevalence STIs. In our sample, the majority of diagnosed cases of gonorrhea or chlamydia and untreated syphilis were asymptomatic, less than half of participants with symptoms sought any form of medical treatment, and one-third of symptomatic participants took no action at all. As a result, the majority of cases of chlamydia in heterosexual women and recent syphilis in MSM in our study population would only have been detected through a program of routine laboratory screening directed towards the specific STIs endemic to these sub-populations. As a result, we would argue that laboratory-based STI screening, guided by individual risk behavior history and regional epidemiologic data on STI prevalence, is an essential complement to syndromic management of symptomatic infections for STI control in developing regions of Latin America.

Our study has several limitations that could have influenced our findings. Our data was collected as part of an HIV/STI prevention trial that was not specifically designed to assess syndromic management as an STI control strategy. Symptomatic infections were identified as those where participants reported symptoms at any time within the previous six months. This definition may have increased the sensitivity of “symptomatic” criteria for STI detection, but limited their specificity for diagnosis. Participants were not physically examined to assess for the presence of clinical signs of infection, and there were no follow-up questions to elaborate on reported symptoms of dysuria, urogenital discharge, or genital ulceration. In particular, the lower prevalence of laboratory-confirmed gonorrhea and chlamydia among symptomatic participants who did not seek treatment compared with those who sought attention for their symptoms suggests the possibility that some of the symptoms reported by this subgroup would have been found to be minimal on further questioning. The reasons why participants did or did not seek treatment were not elicited in our survey and indicate an important area for future research. We are also uncertain of the impact of previous antibiotic treatment on the participant's STI status at enrollment. Appropriate antibiotic treatment may have reduced our estimate of the prevalence of laboratory-confirmed STIs among participants with genitourinary symptoms and, as a result, the estimated specificity of syndromic diagnosis. However, we do not have information on the medication used and cannot determine if the treatment was indicated for the participant's symptoms, appropriate for their syndromic diagnosis, or effective in treating their infection. Finally, we did not assess for rectal or pharyngeal STIs by clinical history or laboratory analysis, leaving an important gap in the knowledge of STI epidemiology in Peru. Despite these limitations, the diagnostic approach and questions used in our study are consistent with typical screening guidelines for the syndromic evaluation of patients at risk for STI acquisition and reflect the impact of a syndromic approach to diagnosis.

As illustrated in our findings, while syndromic management may be an effective method for clinical management of symptomatic STIs, it fails to address the large number of infections that are asymptomatic or otherwise unrecognized by patients, and is inadequate as a primary method for STI control in resource-limited settings. In addition to standard public health measures such as behavioral risk reduction counseling, partner notification and treatment, and condom distribution, laboratory-based screening targeting specific, high-prevalence STIs in at-risk sub-populations provide an important adjunct to syndromic management for STI control efforts in Peru and other developing countries. Further research is needed to determine the cost-benefit ratio of introducing laboratory-based screening into a comprehensive public health program for the control of syphilis, gonorrhea, and chlamydia in populations at risk for HIV and STIs in Latin America.
